# Aberrant transcriptional networks in step-wise neurogenesis of paroxysmal kinesigenic dyskinesia-induced pluripotent stem cells

**DOI:** 10.18632/oncotarget.10680

**Published:** 2016-07-18

**Authors:** Chun Li, Yu Ma, Kunshan Zhang, Junjie Gu, Fan Tang, Shengdi Chen, Li Cao, Siguang Li, Ying Jin

**Affiliations:** ^1^ Laboratory of Molecular Developmental Biology, Shanghai Jiao Tong University School of Medicine, Shanghai, 200025, China; ^2^ Department of Neurology, Ruijin Hospital Affiliated to Shanghai Jiao Tong University School of Medicine, Shanghai, 200025, China; ^3^ Key Laboratory of Stem Cell Biology, Center for The Excellence in Molecular and Cell Sciences, Institute of Health Sciences, Shanghai Institutes for Biological Sciences, Chinese Academy of Sciences and Shanghai Jiao Tong University School of Medicine, Shanghai, 200025, China; ^4^ Stem Cell Translational Research Center, Tongji Hospital, Tongji University School of Medicine, Shanghai 200065, China; ^5^ Collaborative Innovation Center for Brain Science, Tongji University, Shanghai 200092, China

**Keywords:** paroxysmal kinesigenic dyskinesia (PKD), proline-rich transmembrane protein 2 (PRRT2), induced pluripotent stem cells (iPSCs), neural differentiation, transcriptome analysis

## Abstract

Paroxysmal kinesigenic dyskinesia (PKD) is an episodic movement disorder with autosomal-dominant inheritance and marked variability in clinical manifestations.

Proline-rich transmembrane protein 2 (*PRRT2*) has been identified as a causative gene of PKD, but the molecular mechanism underlying the pathogenesis of PKD still remains a mystery. The phenotypes and transcriptional patterns of the PKD disease need further clarification. Here, we report the generation and neural differentiation of iPSC lines from two familial PKD patients with c.487C>T (p. Gln163X) and c.573dupT (p. Gly192Trpfs*8) *PRRT2* mutations, respectively. Notably, an extremely lower efficiency in neural conversion from PKD-iPSCs than control-iPSCs is observed by a step-wise neural differentiation method of dual inhibition of SMAD signaling. Moreover, we show the high expression level of PRRT2 throughout the human brain and the expression pattern of PRRT2 in other human tissues for the first time. To gain molecular insight into the development of the disease, we conduct global gene expression profiling of PKD cells at four different stages of neural induction and identify altered gene expression patterns, which peculiarly reflect dysregulated neural transcriptome signatures and a differentiation tendency to mesodermal development, in comparison to control-iPSCs. Additionally, functional and signaling pathway analyses indicate significantly different cell fate determination between PKD-iPSCs and control-iPSCs. Together, the establishment of PKD-specific *in vitro* models and the illustration of transcriptome features in PKD cells would certainly help us with better understanding of the defects in neural conversion as well as further investigations in the pathogenesis of the PKD disease.

## INTRODUCTION

Paroxysmal kinesigenic dyskinesia (PKD) was initially described in 1967 and is now recognized as the most common type of paroxysmal movement disorder [1-3]. PKD exhibits as an episodic movement disorder with autosomal-dominant inheritance and is characterized by recurrent and brief attacks triggered by sudden voluntary movements [2]. The episodes of movement disorders are usually combined with dystonia, chorea, athetosis or ballism. The clinical manifestations typically occur during childhood or early adulthood with short duration, normal neurologic examination, exclusion of other causes, and symptoms become less severe with age [4-6].

Several linkage studies have identified two regions on chromosome 16 (16p11.2– q12.1 and 16q13–q22.1) as the potential pathogenesis-associated gene loci [7-10]. By combining classic linkage analysis with whole-exome sequencing, mutations in the *PRRT2* gene have been identified as the cause of PKD [11]. This result was rapidly supported by other reports performed in families from different ethnic backgrounds with PKD [12-16]. *PRRT2* is a rarely characterized gene, consisting of four exons, encoding the proline-rich transmembrane protein 2, encompassing 340 amino acids and containing two predicted transmembrane domains [11]. More recently, *PRRT2* mutations were also discovered in Infantile Convulsions and Choreoathetosis (ICCA) [15, 17] and Benign Familial Infantile Epilepsy (BFIE) [15, 18, 19]. Within two years, *PRRT2* mutations have been described in over 330 families from different ethnic backgrounds with PKD, BFIE and ICCA [20, 21]. More than 50 mutation loci were identified in *PRRT2*, and most of them are nonsense, frame-shift and splice site mutations. Several of the mutations have been demonstrated to cause altered cellular localization of PRRT2 proteins [17, 20].

To date, the function of PRRT2 and its potential role in the pathogenesis of PKD have been largely unknown. PRRT2 is expressed throughout the mouse central nervous system (CNS) with high expression levels detected in the cortical layers of the cerebral cortex, basal ganglia and cerebellum [11, 15]. A two-hybrid interactome screen revealed a potential interaction between proteins of PRRT2 and SNAP25 (synaptosome associated protein 25), a constituent of SNARE (soluble N-ethylmaleimide-sensitive factor attachment protein receptor) complexes, which regulates synaptic vesicle membrane docking and fusion, a key process in neuronal exocytosis and neurotransmitter release. The result thus indicated that PKD might associate with synaptic dysfunction [22-25]. Many studies have detected an increased SNAP25 immunoreactivity at the glutamatergic terminals [26-28]. In particular, specific cleavage of SNAP25 by botulinum neurotoxin E (BoNT/E) has been shown to inhibit glutamate release from rat hippocampus glutamatergic neurons [29]. A recent study reported relatively high glutamate levels in the plasma samples of PKD patients, and an increased glutamate level in the culture medium of neurons after infection with shRNA-Prrt2 lentivirus, indicating the involvement of PRRT2 in the release of glutamate [30].

Despite intensive and multiple efforts in the past decades, molecular mechanisms underlying the pathogenesis of PKD still remain a mystery mainly due to the lack of adequate PKD disease-specific models and appropriate approaches to analyze PKD pathogenesis. Recent development in induced pluripotent stem cell (iPSC) technology provides an applicable platform to mimic the pathogenic process [31, 32] and recapitulates the disease in a petri dish, therefore facilitating the investigation of disease-specific phenotypes *in vitro*[33]. The development of RNA sequencing technology and new bioinformatical approaches to deal with big data supplies us with more detailed global gene profiles and more precise measurements compared with traditional microarray, which represents a crucial entry point to elucidate the pathophysiology of this disorder.

In the present study, we generated multiple PKD-iPSC lines from two familial PKD patients with c.487C>T (p. Gln163X) and c.573dupT (p. Gly192Trpfs*8) *PRRT2* mutations, respectively, and established neural differentiation system of the *in vitro* models. We observed that PKD-iPSCs exhibited defects in neural conversion via a step-wise neural induction method, with an extremely low efficiency in generating neural precursor cells (NPCs) compared to control-iPSCs. We detected the expression pattern of PRRT2 in human tissues for the first time, and revealed its high expression level throughout the human brain. In addition, we profiled global transcriptomes of stage-specific PKD cells during neural induction. Gene ontology analysis revealed that differentially expressed genes (DEGs) in normal controls were mostly enriched with terms of neuron differentiation, axon guidance, neuron fate commitment and neuron development, especially at the late stage of neural induction. However, DEGs in PKD cells were mainly involved in definitely different biological processes, including blood vessel development, angiogenesis, bone development and skeletal system development. Furthermore, global transcriptome profiling analysis verified different cell fate determination between PKD-iPSCs and control-iPSCs under the same culture condition. Taken together, our study provides an adequate and convenient platform to analyze the pathogenesis of the PKD disease based on the *in vitro* iPSC model. The illustration of transcriptome signatures and the discovery of gene modules related to PKD cells open new avenues to understand the neural system defect in the PKD disease.

## RESULTS

### PRRT2 are highly expressed in the human brain

**Figure 1:**
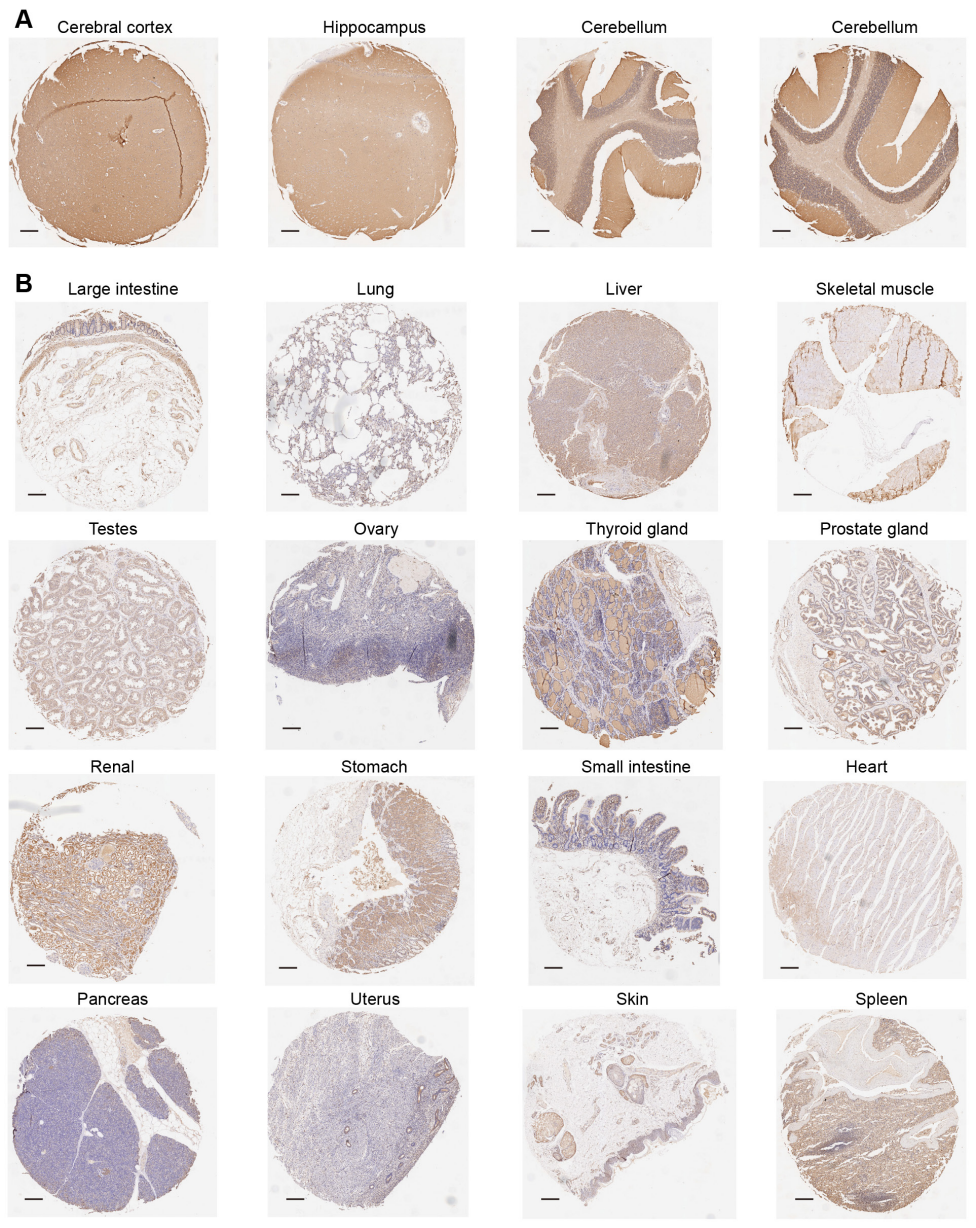
The expression pattern of PRRT2 in the human tissues Tissue microarray analysis was performed to measure the expression pattern of PRRT2 in various adult human tissues. IHC immune-stained sections were scanned using Scanscope XT System (Aperio, Leica). **A.** Immunohistochemistry analysis revealed that PRRT2 was highly expressed throughout the human brain, especially with high levels in the cerebral cortex, hippocampus and cerebellum. **B.** PRRT2 expression patterns in other tissues such as the large intestine, lung, liver, skeletal muscle, testes, ovary, thyroid gland, prostate gland, renal, stomach, small intestine, heart, pancreas, uterus, skin and spleen are shown. Scale bars, 200 μm.

Previous study has reported that PRRT2 was identified as the pathogenesis-associated gene of PKD, and it was highly expressed in the mouse brain and spinal cord, displaying a dynamic expression pattern during mouse development [11]. However, the expression pattern of PRRT2 in human tissues remains unknown mainly due to the lack of effective antibodies against PRRT2. To solve this problem, we developed an affinity-purified polyclonal antibody from anti-human PRRT2 rabbit serum. With the availability of this antibody, we performed tissue microarray to explore the expression pattern of PRRT2 in different adult human tissues. Immunohistochemistry analysis revealed that, in accordance with the finding in the mouse, PRRT2 was highly expressed throughout the human brain, especially in the cerebral cortex, hippocampus and cerebellum, in comparison to other tissues such as the lung, liver, testes, ovary, heart, pancreas, uterus, etc (Figure 1A and 1B). Moreover, we detected the expression pattern in the aborted human fetal brain. Immunofluorescence staining against PRRT2 in human fetal brain slices confirmed the high expression level of PRRT2 in the human fetal brain (Supplementary Figure S1A) and illustrated the plasma membrane localization of PRRT2 proteins (Supplementary Figure S1B). Western blotting also displayed the high expression levels of PRRT2 in different anatomical regions of the human fetal brain (Supplementary Figure S1C). Together, these results indicate that PRRT2 is highly expressed in the human brain.

### PKD-iPSC lines are generated from patient fibroblasts with *PRRT2* mutations

**Figure 2:**
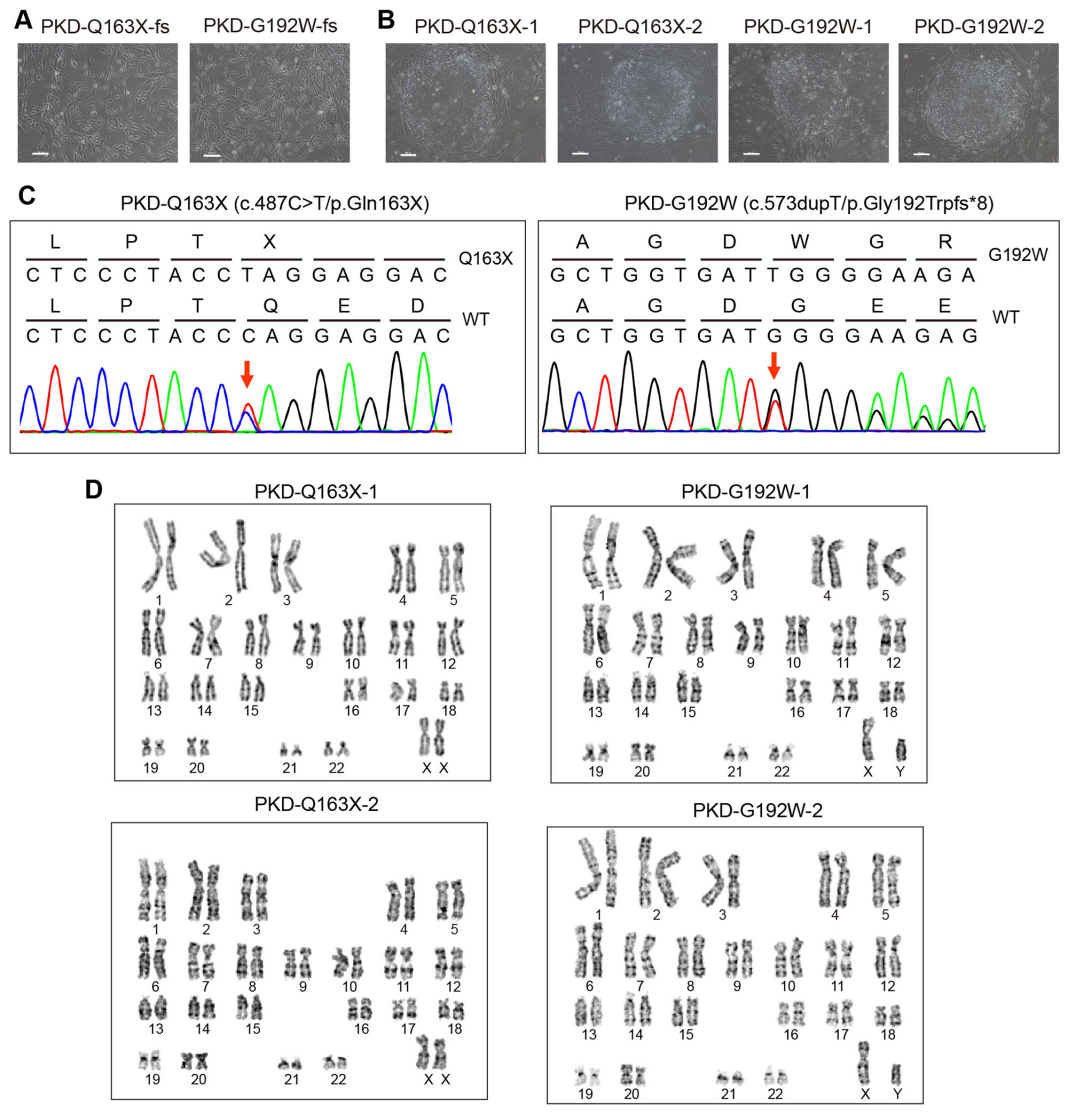
Generation of iPSC lines from fibroblasts (fs) of PKD patients with *PRRT2* mutations **A.** Primary culture of PKD-Q163X-fs and PKD-G192W-fs. Scale bars, 100 μm. **B.** Established iPSC lines (PKD-Q163X-1, 2 and PKD-G192W-1, 2) from PKD patients showed embryonic stem cell-like morphology. Scale bars, 100 μm. **C.** The iPSC colonies derived from PKD-Q163X-fs were verified to carry the heterozygous c.487C>T (p. Gln163X) mutation and iPSC colonies derived from PKD-G192W-fs were verified to carry an inDel c.573dupT (p. Gly192Trpfs*8) via Sanger sequencing. **D.** All iPSC lines derived from the two PKD patients have normal karyotypes.

We established PKD-iPSC lines from dermal fibroblasts of two PKD patients carrying heterozygous *PRRT2* mutations, an inDel c.573dupT (p. Gly192Trpfs*8) in a female patient (named PKD-G192W-fs) and a c.487C>T mutation (p. Gln163X) in a male patient (named PKD-Q163X-fs) (Figure 2A). Fibroblasts were reprogrammed into iPSCs using retroviral vectors encoding Yamanaka factors OSKM (OCT4, SOX2, KLF4 and C-MYC) as previously described [32]. The human embryonic stem cell (hESC)-like colonies were manually picked from day 12 to day 37 of infection and expanded to obtain stable iPSC lines. Two PKD-iPSC lines containing the Q163X mutation (named PKD-Q163X-1, 2) and two cell lines containing the G192W mutation (named PKD-G192W-1, 2) were generated. PKD-iPSCs maintained hESC-like morphology for at least 38 passages (Figure 2B) with the original *PRRT2* mutations throughout the reprogramming process (Figure 2C) and the same normal karyotypes as their cognate fibroblasts (Figure 2D).

**Figure 3:**
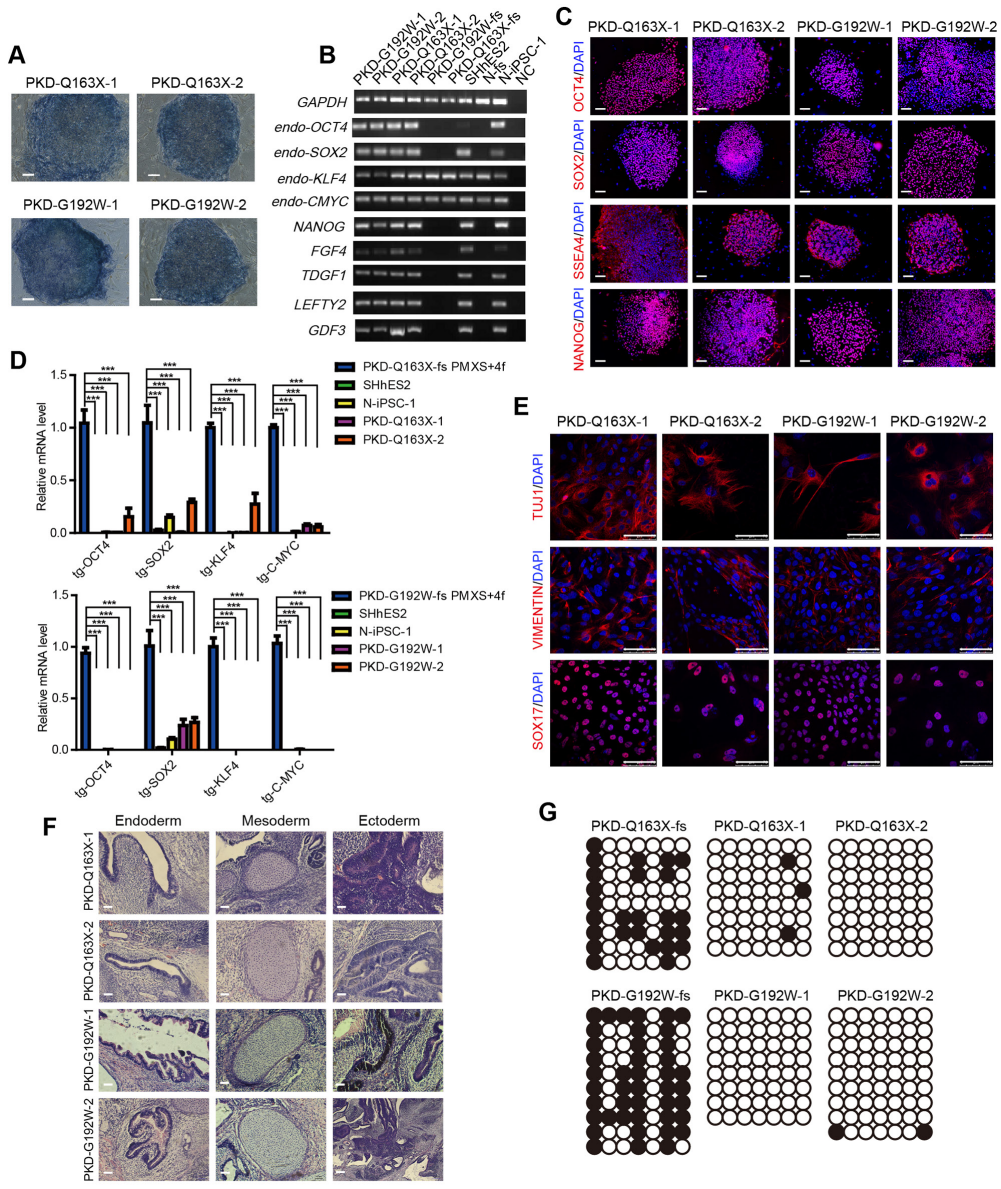
Characterization of iPSCs from PKD patients **A.** Alkaline phosphatase staining of all established iPSC lines. Scale bars, 100 μm. **B.** RT-PCR assays for detecting the expression of pluripotency-associated markers in PKD-Q163X-1, 2 and PKD-G192W-1, 2. **C.** Immunofluorescence staining against pluripotency markers OCT4, SOX2, NANOG and SSEA4 in PKD-Q163X-1, 2 and PKD-G192W-1, 2. Scale bars, 100 μm. **D.** Quantitative RT-PCR analysis of expression levels of 4 transgenic genes *OCT4, SOX2, KLF4* and *C-MYC* in PKD-Q163X-1, 2 and PKD-G192W-1, 2. The expression level of each transgenic gene in PKD-fs infected with retroviruses for 5 days was set as positive control. N-iPSC-1 and SHhES2 served as negative controls. The expression level of transgenes in all PKD-iPSC lines are calculated and shown as mean ± SD. ***p < 0.001. **E.** Immunofluorescence staining of differentiated cells from EBs formed by PKD-Q163X-1, 2 and PKD-G192W-1, 2 using antibodies against SOX17 (endoderm), VIMENTIN (mesoderm) and TUJ1 (ectoderm). Scale bars, 75 μm. **F.** H & E staining images of teratomas formed by PKD-Q163X-1, 2 and PKD-G192W-1, 2. Goblet cells representing the endoderm, the cartilage representing the mesoderm, the neural epithelium and pigment cells representing the ectoderm are shown. Scale bars, 50 μm. **G.** Bisulfite sequencing analysis of endogenous OCT4 promoter methylation in PKD-Q163X-fs, PKD-G192W-fs, PKD-Q163X-1, 2 and PKD-G192W-1, 2. Hollow circles denote unmethylated CpG sites and the black circles denote methylated CpG sites.

Cells of all established iPSC lines were alkaline phosphatase positive (Figure 3A), and endogenously expressed OCT4, SOX2, KLF4 and C-MYC, as well as other pluripotency-associated markers such as NANOG, FGF4, TDGF, LEFTY, GDF3 and SSEA4 (Figure 3B and 3C), while the expression of transgenic genes was silenced significantly in these iPSC cell lines (Figure 3D). Additionally, we examined the differentiation potential of iPSCs through both *in vitro* and *in vivo* assays. Embryoid body (EB) formation assays revealed the expression of all three germ layer markers, such as SOX17 (endoderm), VIMENTIN (mesoderm) and TUJ1 (ectoderm) (Figure 3E). Simultaneously, development of teratomas containing the respiratory epithelium and goblet cells (endoderm), muscles and cartilages (mesoderm), neural rosette and pigmented cells (ectoderm) confirmed the derivatives of all three germ layers (Figure 3F). Moreover, similar to hESCs, the OCT4 promoter was hypomethylated in all iPSCs, but hypermethylated in their cognate fibroblasts, further verifying the reprogramming of fibroblasts (Figure 3G).

### PKD-iPSCs exhibit severe defects in neural differentiation through a step-wise neural induction process

**Figure 4:**
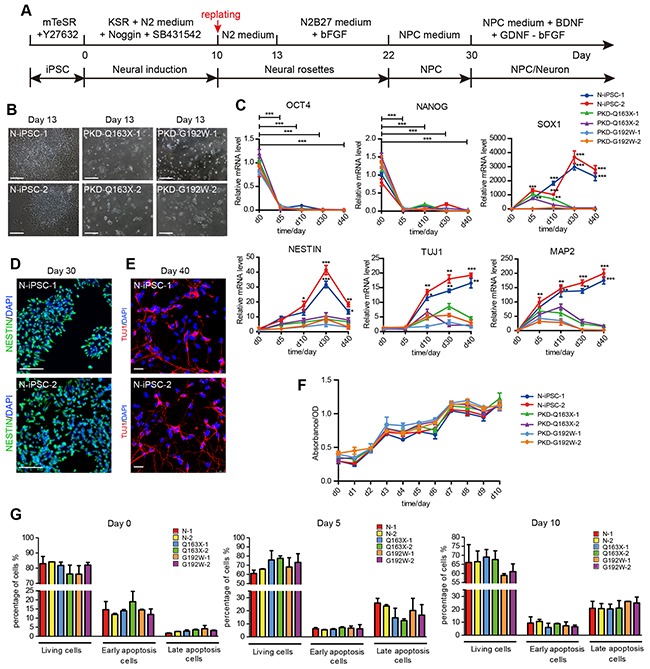
A step-wise neural induction of iPSC lines from PKD patients and a control individual **A.** Flow diagram of the neural induction process. **B.** Normal iPSC lines (N-iPSC-1, 2) showed neural rosette-like morphology. PKD-iPSC lines (PKD-Q163X-1, 2 and PKD-G192W-1, 2) showed severe defects in forming neural rosettes. Scale bars, 100 μm. **C.** Quantitative RT-PCR analysis revealed similar expression levels of pluripotency markers (OCT4 and NANOG), but obviously different expression patterns of NPC markers (SOX1 and NESTIN) and neuron markers (TUJ1 and MAP2) between PKD and normal groups. The expression level of marker genes in all PKD-iPSC lines are calculated and shown as mean ± SD. *p < 0.05; **p < 0.01; ***p < 0.001. **D.** Immunofluorescence staining against NPC marker (NESTIN) in N-iPSC-1, 2 on day 30. Scale bars, 100 μm. **E.** Immunofluorescence staining against neuron marker (TUJ1) in N-i-1, 2 on day 40. Scale bars, 25 μm. **F.** The cell growth curve detected in all iPSC lines from day 0-10 of neural induction displayed similar cell growth levels. The absorbance was measured at 450 nm and 600nm. The optical density (OD) were calculated and shown as mean ± SD. **G.** Comparable levels of cell apoptosis were detected in normal iPSCs and PKD-iPSCs on days 0, 5 and 10, respectively. Levels of cell apoptosis were analyzed by Annexin V-PE/7-AAD staining, followed by flow cytometry analysis. Quantification of apoptosis parameters is shown. Student's t-test was used to test whether there is significant difference between normal and PKD groups. Error bars represent standard errors.

To determine whether PKD-iPSCs display altered neural differentiation, we generated a relatively homogenous population of NPCs from both PKD-iPSCs and normal iPSCs basing on a previously published method for step-wise adhesive monolayer neural induction of human pluripotent cells through the synergistic action of two inhibitors of SMAD signaling (Noggin and SB431542) [34] with minor modifications [35]. At day 10 post-induction, cells were digested into small clumps for the first replating. NPCs were obtained within one month, and subsequently cultured in the medium containing neurotrophic factors that promoting spontaneous neuronal differentiation (Figure 4A). Here, two lines of iPSCs from a normal unrelated individual (N-iPSC-1, 2) established previously in our lab [36] served as the non-affected controls. Normal iPSCs efficiently formed neural rosettes (Figure 4B) and further expanded as NPCs in the presence of bFGF. During the neural differentiation process, the pluripotency-associated factors OCT4 and NANOG in all iPSC lines were rapidly silenced. For normal iPSCs, the neural lineage markers NESTIN and TUJ1 were activated and their expression levels gradually increased along the differentiation course (Figure 4C-4E). Remarkably, in contrast to the high efficiency in neural conversion from normal iPSCs, PKD-iPSCs exhibited significant impairment in the neural differentiation under the same induction condition. Majorities of PKD-iPSCs lost the survival abilities after the first replating and therefore encountered difficulties to develop into neural rosettes (Figure 4B). Quantitative RT-PCR results revealed that markers indicative of neural lineages (SOX1, NESTIN, TUJ1and MAP2) were expressed at extremely lower levels in cells derived from PKD-iPSCs than in the normal controls (Figure 4C). Thus, our results uncover notable defects in neural differentiation of PKD-iPSCs in the step-wise neural induction process.

Since we observed a sharp decrease of differentiated neural cells from PKD-iPSCs after the first replating during neural differentiation, the cellular growth and apoptosis rates were examined to address the question of whether these defects in neural differentiation were related to cell proliferation and apoptosis before replating. We measured the cell growth during the early stage (before replating, day 0-10) of neural induction and evaluated the apoptosis at different time points (before replating, days 0, 5 and 10) of neural induction. Interestingly, normal and PKD cells displayed comparable rates of proliferation and apoptosis before the first replating procedure (Figure 4F and 4G), thus excluding the possibility that the alteration in neural differentiation is caused by changes in the proliferation and apoptosis.

As PKD patients do have fully developed brains, the severe defects in neural conversion of PKD-iPSCs prompted us to question whether PKD-iPSCs tend to undergo different cell fate commitment at different culture conditions. To test this assumption, we applied a rapid single-step neural induction method using the PSC Neural Induction Medium provided by Life Technologies Corporation (A1647801), to induct neural differentiation of iPSCs. Using this method, PKD-iPSCs as well as normal iPSCs could be converted into the neural lineage with high efficiencies. NESTIN and SOX1 positive cells (Neural stem cells, NSCs) were obtained in one week, and TUJ1/MAP2 positive cells (neurons) appeared within two weeks (Supplementary Figure S2). In brief, in contrast to the severe phenotypes in neural conversion by the step-wise method using dual inhibition of SMAD signaling, PKD-iPSCs went smoothly to the neural lineage when the single-step neural induction method was utilized. Hence, it is likely that different neural induction approaches may bring out different differentiation process and cell fate tendency. We can choose different neural induction methods depending on different research purposes. In this study, we focus on the detailed differences of cell phenotype and transcriptional expression profiles between PKD-iPSCs and normal iPSCs during the relatively early phases of neural differentiation, which could be captured through the step-wise neural induction method by dual inhibition of SMAD signaling.

### PKD-iPSCs have aberrant gene expression profiles and biological processes during a step-wise neural differentiation

**Figure 5:**
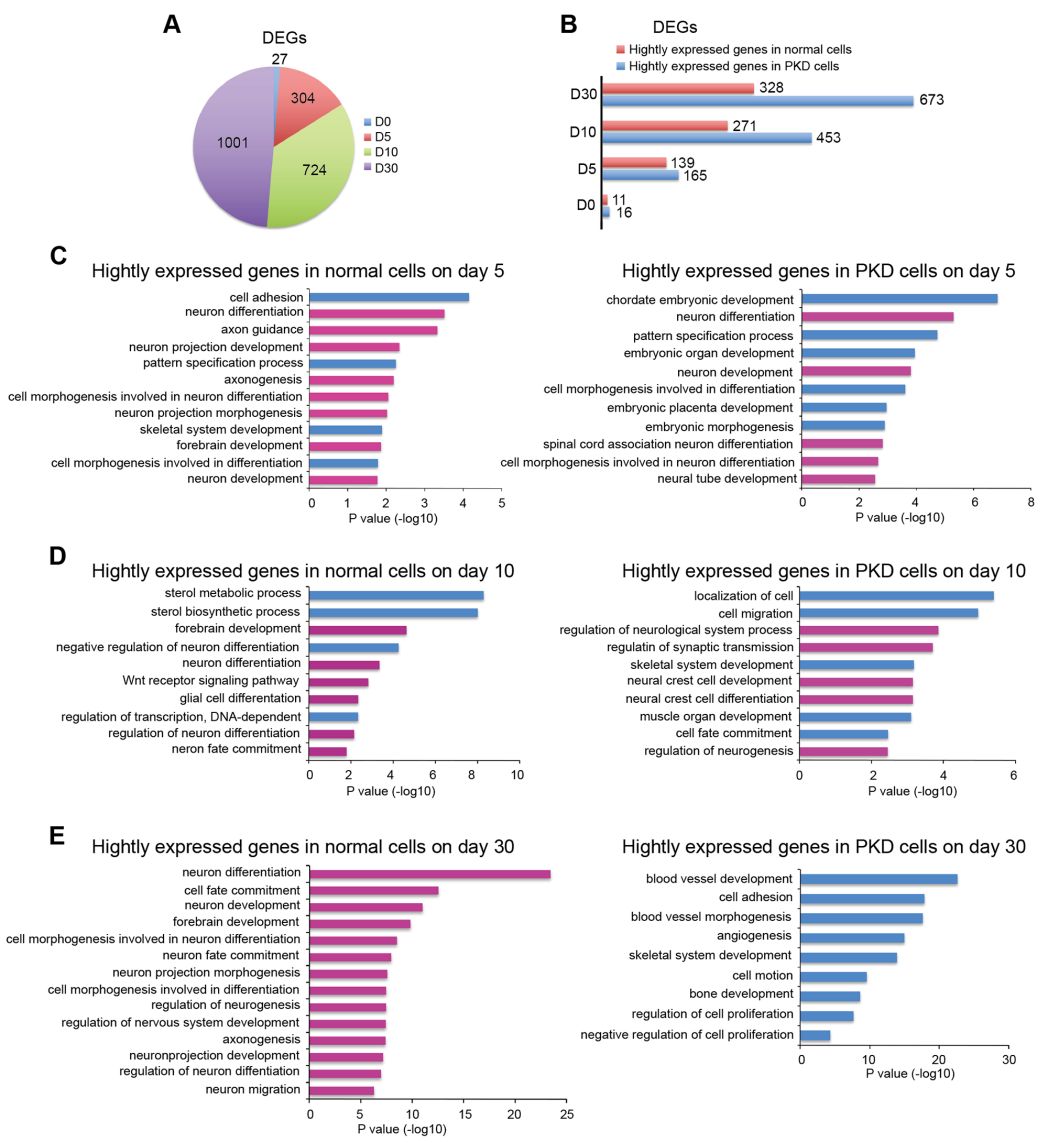
Gene expression profile analysis of DEGs between PKD-iPSCs and normal controls during a step-wise neural differentiation **A.** Pie chart shows the number of differentially expressed genes (DEGs, FC ≥ 2 or FC ≤ 0.5, P value < 0.05, FC=Fold Change.) at different time points of neural differentiation. **B.** Distribution of the DEGs exhibits in PKD cells and normal cells on days 0, 5, 10 and day 30. **C-E.** The Gene Ontology (GO) analysis of highly expressed DEGs in PKD cells and normal cells on day 5 (C), day 10 (D) and day 30 (E) of neural induction. The top significant GO categories identified in each of the two groups are shown. Pink-colored GO terms are associated with neurogenesis; blue-colored GO terms are associated with other biological processes exclusive of neural development. The length of bars indicates –log_10_ P value of the Fisher's exact test.

To gain molecular insights into how the pathogenic *PRRT2* mutations caused neurogenic defects, we compared transcriptomes of PKD cells and normal controls at different stages of the step-wise neural differentiation. Cells from both PKD-iPSCs and normal iPSCs, separately collected on days 0, 5, 10 and 30 of neural induction, were subjected to RNA-seq sample preparation, cDNA library construction and then deep sequencing. Subsequently, RNA-seq data were mapped to the human genome assembly hg19. The mapping rates reached to > 94% for each sample. A total of 2056 differentially expressed genes (DEGs) between PKD and normal cells were identified (Figure 5A), of which 1307 genes were highly expressed in PKD cells and 749 genes were highly expressed in normal cells (Figure 5B). The numbers of DEGs enlarged from day 0 to day 30 (Figure 5A), indicating the gradually increased deviation in the gene expression profiles of PKD cells from normal cells along neural differentiation.

These DEGs were supplied to gene ontology (GO) enrichment analysis in a time-dependent manner. The results revealed striking features of these DEGs. First, at the early stages (days 5 and 10) of neural differentiation, the enriched biological processes revealed relatively little difference between PKD and normal cells (Figure 5C and 5D). Second, extremely different gene expression profiles appeared on day 30. The top five significantly enriched GO categories in normal cells were “neuron differentiation”, “cell fate commitment”, “neuron development”, “forebrain development” and “cell morphology involved in neuron differentiation”, all of which are associated with neurogenesis (Figure 5E). In contrast, PKD cells gradually lost the biological processes related with neural cell fate commitment. The top five significantly enriched GO categories in PKD cells were “blood vessel development”, “cell adhesion”, “blood vessel morphogenesis”, “angiogenesis” and “skeletal system development” (Figure 5E). Therefore, our results indicate that PRRT2 might play an important role for the neural induction in human pluripotent stem cells.

### PKD-iPSCs display dysregulated neural transcriptome signatures during a step-wise neural induction

**Figure 6:**
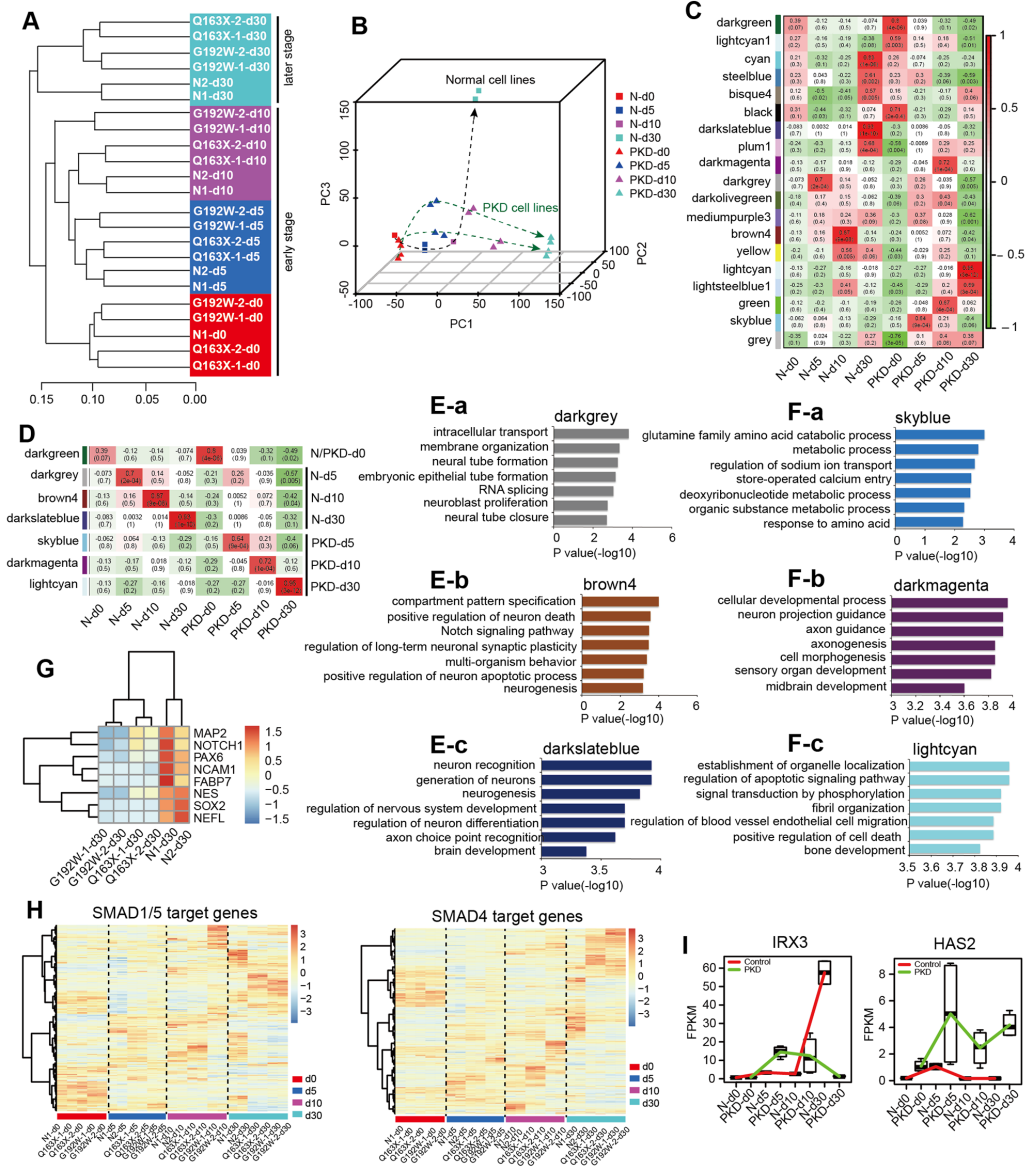
Altered global transcriptomes and dysregulated neural transcriptomes in PKD cells during a step-wise neural induction **A.** Unsupervised hierarchical clustering of the transcriptomes of all samples. **B.** Principal component analysis (PCA) of PKD and normal samples from four stages of neural differentiation. Cells with same sample types are shown in the same shape. Cells in same time point are shown in the same color. Dash lines with an arrow indicate the developmental direction of each cell line. **C-D.** Stage-specific co-expression gene modules identified by WGCNA and their correlation to the development stage are shown in (C). The number of each square represents a correlation of modules and sample types, and p-value of each correlation represents a correlation value. The color of each square is corresponding to the correlation: Positive correlation (Red); Negative correlation (Green); No correlation (White). Modules with highest correlation to each sample type are shown in (D). **E-F.** The top seven significant GO categories identified in modules with highest correlation are shown. Darkgrey module **E-a.** brown4 module **E-b.** and darkslateblue module **E-c.** are related with normal samples N-d5, N-d10 and N-d30, respectively. Skyblue module **F-a.** darkmagenta module **F-b.** and lightcyan module **F-c.** are related with PKD samples PKD-d5, PKD-d10 and PKD-d30, respectively. The length of bars indicates –log_10_ P value of the Fisher's exact test. **G.** Expression patterns of neural lineage markers for samples collected on day 30. Colors represent gene expression levels: High expression (Red); Moderate expression (White); Low expression (Blue). **H.** Heatmaps illustrate expression values of SMAD1/5 and SMAD4 target genes in all samples. **I.** Boxplots indicate the distribution of *IRX3* and *HAS2* expression in different samples. The black line in boxplots refers to the median. The red line represents the control group; the green line represents the PKD group.

To verify the properties and relationship of each cell sample described above, we performed unsupervised hierarchical clustering [37] and Principal-Component Analysis (PCA) [38]. Unsupervised hierarchical clustering of PKD and normal cell samples from different neural differentiation stages (days 0, 5, 10 and 30) showed that these samples clustered together naturally depending on developmental stages (Figure 6A). Moreover, the developmental order was accurately categorized from early stage (days 0, 5 and 10) to late stage (day 30) of the neural inducing process, as neighboring time points clustered together in the analysis (Figure 6A). Furthermore, similar within-stage and different between-stage expression patterns were also supported by PCA (Figure 6B). PCA addressed different attributes among these cell samples, which were definitely divided into two distinct groups, indicating the different properties and cell fate decisions between PKD cells and normal cells in the same neural induction system (Figure 6B).

To investigate the molecular features of PKD derived cells and the pathogenesis of PKD, we performed Weighted Gene Co-expression Network Analysis (WGCNA) [39] for detecting gene co-expressed modules of PKD and normal cells along the neural inducing process (Supplementary Figure S3A). Nineteen co-expression gene modules were identified based on time points and sample types (N-d0, N-d5, N-d10, N-d30, PKD-d0, PKD-d5, PKD-d10 and PKD-d30) (Figure 6C). Notably, there were seven modules (darkgreen, darkgrey, brown4, darkslateblue, skyblue, darkmagenta and lightcyan) with the highest correlation to each group (Figure 6D). These modules were analyzed according to normal and PKD groups. Functional analysis of these gene modules indicated that neural lineage related GO terms were gradually enriched from day 5 to day 30 in normal groups (Figure 6E-a, -b, -c). On the contrary, in PKD groups, there was a delay of GO terms related with neurogenesis that enriched on day 10 (Figure 6F-b). Notably, none of these neurogenic categories maintained to day 30 (Figure 6F-c), which was consistent with the defects in neural conversion of PKD cells (Figure 4B). Surprisingly, GO categories related to mesodermal development such as “regulation of blood vessel endothelial cell migration” and “bone development” were enriched in PKD groups on day 30 (Figure 6F-c). Some neural lineage markers such as MAP2, NOTCH1, PAX6, NCAM1, FABP7, NESTIN, SOX2 and NEFL were lowly expressed in PKD samples, while highly expressed in normal samples, further supporting the idea that PKD cells underwent completely different cell fate commitment, other than neural development (Figure 6G). Similar results were obtained using Kyoto Encyclopedia of Genes and Genomes (KEGG) analysis (Supplementary Figure S3B and S3C). Together, these analyses revealed dysregulated neural transcriptomes of PKD-iPSCs in neural induction, which might hinder PKD-iPSCs from differentiating into neural cell lineages and simultaneously induce the fate of mesodermal lineages.

Since a delay of GO terms related with neurogenesis appeared from early differentiation stages, we hypothesized that the molecular events leading to the neural conversion defect may initiate from day 0 to day 10, when cells were cultured with the medium containing two inducing factors Noggin and SB431542. To test this, we examined the expression patterns of SMAD1/4/5 target genes [40] in all samples, as Noggin and SB431542 synergistically promoted neural conversion of iPSCs through blocking the SMAD signaling pathway [34]. Different expression patterns were detected between PKD cells and normal controls throughout the neural differentiation stages (days 5, 10 and 30), and the difference increased along the time-course, although the majority of SMAD target genes presented the similar expression level before neural induction (day 0) (Figure 6H). Target genes expressed at relatively high levels for PKD and normal groups from each differentiation stage were submitted to the DEGs datasets. Among these targets, *IRX3* (iroquois homeobox 3) and *HAS2* (Hyaluronan Synthase 2) were found to be differentially expressed at all stages (Figure 6I). IRX3 is a target of SMAD1/5 and plays a key role in the early stage of neuronal differentiation [41, 42]. HAS2, a synthase of Hyaluronic Acid (HA), is a target gene of SMAD4 and serves as a member of the BMP-SMAD signaling pathway [43]. Several reports indicated that HAS2 is critical for mesodermal commitment such as limb development, vessel development, bone development, cartilage development [44-46], and is also required for myogenic differentiation [47, 48]. In normal controls, the synergistic effect of a sharply enhanced expression of IRX3 from day 10 and a continuously quite low level of HAS2 could facilitate the neural differentiation. However, in comparison to normal controls, drastically different expression patterns of both IRX3 and HAS2 were manifested in PKD cells, which might be implicated in the dysregulation of neurogenesis in PKD-iPSCs (Figure 6I).

Additionally, through the measure of intramodular gene connectivity (kME), we identified intramodular hub genes in the stage-specific modules. Intramodular hub genes are centrally located in their respective modules and may thus be critical components within the gene network. We used the VisANT [49] to visualize the top 100 gene connections (based on topological overlap) among the top 100 intramodular hub genes. Gene networks of both PKD and normal groups indicated that PKD-iPSCs exhibited a differentiation tendency towards mesodermal development, while normal iPSCs had an access to neural conversion (Supplementary Figure S3D and S3E), which was consistent with the results described above (Figure 6E-6G, Supplementary Figure S3B and S3C). Although we could not yet put forward a solid basis of a theory to thoroughly elucidate the pathogenesis of PKD, investigation of the transcriptome signatures and discovery of the gene modules highly related with the pathogenesis of PKD would help us with better understanding of the potential causes leading to abnormal differentiation of PKD-iPSCs.

## DISCUSSION

PKD is a remarkable hereditary disorder and many genetically proven PKD clinical cases have been published to date [50-52]. *PRRT2* was first identified as a causative gene of PKD in two Chinese studies [11, 12], and then supported by many researches on PKD studies. A recent study measured the expression levels of PRRT2 in mouse tissues, and revealed its high expression levels throughout the mouse brain compared to heart, lung, kidney and skin [11]. In this study, we developed a highly effective and specific human PRRT2 antibody, which allowed us to demonstrate that the expression level of PRRT2 was much higher in the human brain than in other human tissues, thus providing experimental evidence for a potential relationship between *PRRT2* and neurogenesis. To the best of our knowledge, this study is the first to report the expression pattern of PRRT2 in human tissues.

Despite intensive and multiple efforts, the cellular and molecular mechanisms underlying the pathogenesis of PKD remain an open question. The lack of appropriate PKD patient-specific models becomes one of the most important restrictions for further investigation in PKD study. To overcome this barrier, we established PKD-specific iPSC lines as *in vitro* models. In this study, we mimicked the neurogenesis of the PKD disease using these iPSC models. PKD-iPSCs were induced into neural lineage cells in an optimized differentiation system using a step-wise neural induction approach. Remarkably, we observed severe defects in neural conversion of PKD-iPSCs compared with normal controls, showing great difficulties to form neural rosettes. This finding raises the possibility that PRRT2 plays an important role for early neural induction, although the relevance of phenotypes observed in PKD-iPSCs to the pathology of the PKD disease remains unknown.

The development of RNA-Seq techniques and new bioinformatical approaches supplies us with more detailed global transcriptomes and more precise results, making it feasible to comprehensively define the molecular features of PKD cells. Via these approaches, we found dysregulated neural transcriptome signatures in PKD cells, with a lack of molecular properties relative to neural differentiation, showing the deficiency in generating neural lineage cells for PKD-iPSCs. Additionally, functional analysis as well as signaling pathway analysis revealed extremely different cell fate commitment between PKD-iPSCs and normal iPSCs. Gene networks of both PKD and normal groups indicated that PKD-iPSCs exhibited a differentiation tendency to mesodermal development, while normal iPSCs were in preference to neural conversion under the same induction condition. Furthermore, the discovery of SMAD target genes *IRX3* and *HAS2*, which differentially expressed in PKD and normal groups, provides us with a reasonable clue accounting for the pathological process of PKD.

In resent decades, high-throughput sequencing has identified mutations in the *PRRT2* gene as the leading cause for varieties of paroxysmal diseases besides PKD and > 50 different *PRRT2* mutations have been documented [20, 53, 54]. To date, heterozygous *PRRT2* mutations have been found in a multitude of patients with BFIE [20], ICCA [20, 53], Paroxysmal Non-Kinesigenic Dyskinesia (PNKD) [55], Paroxysmal Exertion-induced Dyskinesia (PED) [56], childhood absence epilepsy and febrile seizures [57, 58]. Besides, homozygous *PRRT2* mutations have been mainly associated with intellectual disabilities [20, 54]. Markedly, these diseases corporately share clinical manifestations with PKD. Therefore, *PRRT2* is a gene with remarkable pleiotropy, and uncovering the function of PRRT2 in PKD can serve to elucidate the underlying mechanisms for a broad range of paroxysmal movement disorders.

Overall, our study provides applicable PKD-specific *in vitro* models and a novel insight into the particular transcriptome features in PKD-iPSCs derived cells, thus facilitating future investigations in PKD as well as other paroxysmal movement disorders.

## MATERIALS AND METHODS

### Tissue microarray analysis

Adult human tissue microarrays were provided by the Shanghai Outdo Biotech Company. Information of some tissues on the microarray is listed in Table 1. In brief, immunohistochemical (IHC) staining was performed using tissue microarrays which incubated in a dry oven at 63 °C for 1 hour. Formalin-fixed, paraffin-embedded sections were deparaffinized and rehydrated. After antigen retrieval and blocking, sections were incubated overnight at 4 °C with primary antibody anti-PRRT2. Then secondary antibody incubation was carried out at room temperature for 30 minutes, followed by treatment with DBA solution. Hematoxylin staining was applied to sections for the nuclei detection. IHC immune-stained sections were scanned using the Scanscope XT System (Aperio, Leica) that could continuously create complete digital slides and deliver volumes of digital slides with superior image quality.

**Table 1:** Information for adult human tissues

Specimen No.	Tissue type	Sex	Age
1	Cerebral cortex	Male	25
2	Hippocampus	Male	25
3	Cerebellum	Male	25
4	Cerebellum	Male	25
5	Large intestine	Male	48
6	Lung	Female	20
7	Liver	Male	47
8	Skeletal muscle	Male	45
9	Testes	Male	25
10	Ovary	Female	23
11	Thyroid gland	Male	25
12	Prostate gland	Male	25
13	Renal	Male	42
14	Stomach	Male	60
15	Small intestine	Female	23
16	Heart	Male	25
17	Pancreas	Female	40
18	Uterus	Female	36
19	Skin	Male	45
20	Spleen	Male	45

### Human PRRT2 antibody preparation

We chose the fragment containing amino acids 1-191 from extracellular domain of *PRRT2* gene as the human specific PRRT2 epitope. The primers designed to amplify the PRRT2 antigen coding sequence are as follows: CCGGGATCCA TGGCAGCCA GCAGCTCT (forward primer, 5’ to 3’); CCCCTCGAGCTAGGTAGGGAGCTCTG GTTGA (reverse primer, 5’ to 3’). PCR products containing the PRRT2 antigen were cloned into the pGEX-4T-1 vector (GE Healthcare Life Sciences) which contains Glutathione S-Transferase (GST) coding sequences. The GST-antigen fusion proteins were purified through Glutathione-Sepharose 4B beads (GE Healthcare Life Sciences) and eluted with the elution buffer consisting of 50 mM Tris-HCl, 4 M NaCl and 10 mM glutathione (Sigma). The purified GST-antigen fusion proteins were used to immunize two rabbits for three times and these immune rabbit serums were collected at 2-3 months after the third immunization. Further purification of antigen was performed with affinity chromatography for the human PRRT2 polyclonal antibody.

### Derivation of PKD patient fibroblasts

PKD patient human dermal fibroblasts were generated from explants of dermal biopsies, which were obtained from the Ruijin Hospital at the approval of the research ethics committee of the hospital after getting the informed consents from the donors. Fibroblast outgrowths from the explants were passaged and cultured in the epithelial cell growth medium as previously described [59].

### Generation and culture of PKD patient-specific iPSCs

Retroviruses carrying human OCT4, SOX2, KLF4 and C-MYC (OSKM) coding sequences were prepared as previously reported [32, 59, 60]. The OSKM were transfected into human fibroblasts at a multiplicity of infection (MOI) of 10 with 20 ng/ml polybrene per 1X10^5^ cells. At 24 hours post-transduction, cells were digested into single cells and replated onto gelatin-coated dishes. The epithelial cell growth medium was replaced by the human iPSC culture medium (hPSM) from the next day. hESC-like colonies emerged and were manually picked from 2-6 weeks for further expansion and characterization. iPSCs (passage ≤ 25) were cultured on a feeder layer of irradiated mouse embryonic fibroblasts (MEFs) in hPSM consisting of KO-DMEM (GIBCO), 20% Knockout Serum Replacement (KSR, GIBCO), 2 mM L-glutamine (GIBCO), 100 mM MEM NEAA (GIBCO), 100 U/ml penicillin, 100 μg/ml streptomycin (Sigma), 0.1 mM β-mercaptoethanol (Sigma), and 4 ng/ml human basic FGF (bFGF, R&D). iPSC colonies were passaged by collagenase IV (5 mg/ml, Invitrogen) or the mechanical method every 4-5 days. iPSCs (passage > 25) were cultured under the feeder-free condition. Cells were cultured on Matrigel (BD Biosciences) with the mTeSR1 medium (Stem Cell Technologies), which was changed daily. iPSCs were passaged by collagenase IV or ReLeSR (Stem Cell Technologies).

### Neural differentiation of PKD-iPSCs by a step-wise induction approach

A step-wise neural induction method [34] with minor modifications [35] was applied to neural differentiation of human iPSCs. PKD-iPSCs and control-iPSCs were initially cultured in the mTesR1 medium as described [61], then disaggregated using Accutase (Life Technologies) for 5-10 minutes, and plated on Matrigel-coated dishes with the mTesR1 medium containing ROCK-Inhibitor (Y-27632, 10 μM; Millipore) at a density of 20,000 cells/cm^2^. Subsequently, cells were cultured to expand in the medium without Y27632 until they were nearly confluent. The initial differentiation medium included the knockout serum replacement (KSR) medium together with 500 ng/ml Noggin (R&D) and 10 μM SB431542 (Tocris Bioscience). During day 5-10 of differentiation, increasing amount of N2 medium (25%, 50%, 75%) was added to the KSR medium every 2 days with the maintaining of 10 μM SB431542 and 500 ng/ml Noggin. At 10 days post-induction, SB431542 and 500 ng/ml Noggin were removed, and then cells were digested into small clumps for the first replating. Later, we picked up rosette-like clumps and plated them onto Matrigel-coated dishes. Cells were dissociated into single cells by 0.05% trypsin (GIBCO). All later passages of NPCs were maintained in the N2B27 medium supplemented with 10 ng/ml of bFGF. On day 30 of differentiation, spontaneously neuronal differentiation was induced by a culture medium consisting of the Neurobasal Medium (GIBCO) supplemented with 2 mM L-glutamine, B27 (Life Technologies), 10 ng/ml BDNF (R&D) and 10 ng/ml GDNF (R&D).

### Neural differentiation of PKD-iPSCs by a rapid single-step induction approach

A rapid single-step neural induction method using the PSC Neural Induction Medium provided by Life Technologies Corporation (A1647801) was carried out to induct neural differentiation of iPSCs. PKD-iPSCs and control-iPSCs were initially cultured in the mTesR1 medium and dissociated into cell clumps with 15-20% confluence. Then cells were cultured with complete PSC Neural Induction Medium consisting of Neurobasal Medium and Neural Induction Supplement for 6 days. The serum-free PSC Neural Induction Medium can initiate neural conversion from human PSCs to NSCs in one week with high efficiency. On day 7 of neural induction, NSCs (P0) were ready to be harvested by being dissociated into a single cell suspension using Accutase and re-suspended with complete Neural Expansion Medium consisting of 49% Neurobasal Medium, 49% Advanced DMEM/F-12 and 2% Neural Induction Supplement. NSCs reached confluence on days 4-6 after replating and could be further expanded in the complete Neural Expansion Medium.

### Genome-wide transcriptome profiling analysis

### RNA-Seq library construction and sequencing

Both PKD patient-specific iPSCs and control-iPSCs were cultured under the condition of step-wise neural induction, and they were separately collected on days 0, 5, 10 and 30. Total cellular RNA was extracted by the Trizol Reagent and RNA-seq libraries were constructed by the Beijing Genomics Institute (BGI; Shenzhen, China). Libraries were sequenced on the Illumina HiSeq2000. Single-end reads of 50 bp length were obtained.

### Reads mapping

All reads were aligned to human genome (hg19) by the spliced read aligner Tophat (version 1.4.1) as described previously [62] while supplying splice junctions annotated in the Refseq and ENSEMBL set of transcript models. FPKMs for the annotation were obtained using Cufflinks (version 2.0.2) with default settings.

### Weighted gene co-expression network analysis (WGCNA) and module detection

“WGCNA” in R package was used to construct the weighted gene co-expression network [63]. A signed weighted correlation network was constructed by first creating a matrix of pairwise correlations between all pairs of genes across the measured samples [64]. Next, the adjacency matrix was constructed by raising the co-expression measure to power β = 22. To minimize the noise and spurious associations, the adjacency matrix was transformed to topological overlap matrix (TOM), which is a robust and biologically meaningful measure of network interconnectedness [65]. Genes with highly similar co-expression relationships were grouped together by performing average linkage hierarchical clustering on the topological overlap. We applied the Dynamic Hybrid Tree Cut algorithm [66] with default parameters to cut the hierarchical tree, and defined modules as branches from the tree cutting. We summarized the expression profile of each module by representing it as the first principal component (referred to as module eigengene) that could explain the most variation of the module expression levels. Modules whose eigengenes were highly correlated (correlation > 0.75) were merged together.

### Identification and visualization of hub genes

Module eigengenes lead to a natural measure of module membership. The correlation of module membership can be quantified by naturally standardizing the interval from -1 to 1 and the corresponding statistical significance measure (P value) can be easily computed. Genes with highest module membership values are referred to as intramodular hub genes. Intramodular hub genes are centrally located inside the module and represent the expression profiles of the entire module [67]. We used VisANT [49] to visualize the top 100 gene connections (based on topological overlap) among the top 100 hub genes.

### Functional enrichment analysis

Two enrichment analyses were performed on the genes of interest by assessing enriched GO categories and enriched KEGG pathways. Functional annotation was performed using the Database for Annotation, Visualization and Integrated Discovery (DAVID, http://david.abcc.ncifcrf.gov/) Bioinformatics Resource [68].

### Statistical analysis

For all experiments, statistical significance was assessed by a standard Student's t test (two-tailed) and error bars represent the standard deviation between three biological replicates. A P value < 0.05 is considered statistically significant.

### Accession number

The GEO accession number for the RNA-seq data and processed files reported in this paper is GEO: GSE83256.

## SUPPLEMENTARY MATERIALS AND METHODS


