# Supplementary material for: Aberrant transcriptional networks in step-wise neurogenesis of paroxysmal kinesigenic dyskinesia-induced pluripotent stem cells
Source: Oncotarget. 2016 Jul 18;7(33):53611–27. doi: 10.18632/oncotarget.10680 (PMC5288209; doi:10.18632/oncotarget.10680)
Supplement: Supplementary file 1 [file oncotarget-07-53611-s001.pdf]

Aberrant transcriptional networks in step-wise neurogenesis of paroxysmal kinesigenic dyskinesia-induced pluripotent stem cells

SUPPLEMENTARY MATERIALS AND METHODS

Characterization of the PKD-iPSC lines

Genotyping

Genomic DNA of fibroblasts and iPSCs was extracted by GenElute™ Mammalian Genomic DNA Miniprep Kits (Sigma) according to the manufacturer's protocol. A pair of specific primer was used to amplify the region around the proline-rich domain of *PRRT2*. PCR products were purified and prepared for sequencing in the Rui Di Company (Shanghai, China).

G banding analysis

Cells cultured with 100 ng/ml colchicine (Sigma) for 8-10 hours were digested into single cells by 0.25% trypsin-EDTA (GIBCO), then suspended in hypotonic solution including 0.16 g potassium chloride and 0.125 g sodium citrate dissolved in 50 ml deionized water. After incubation at 37°C for 30 minutes, cells were fixed by glacial acetic acid and methyl alcohol in 1:3 ratio for 30 minutes. The karyotypes were analyzed in the Da An Company (Shanghai, China).

Alkaline phosphatase (AKP) staining

iPSC colonies were fixed with 4% PFA (Sigma) and permeabilized by 0.2% Triton X-100 (Sigma). The alkaline phosphatase activity was detected using an AKP staining solution as instructed by the manual of Alkaline Phosphatase Substrate Kit III (Vector Laboratories, Inc).

Immunofluorescence staining

Immunofluorescence staining was performed as previously described [1]. Cells were fixed with 4% PFA for 15 minutes, permeabilized by 0.2% Triton X-100 for 15 minutes, and blocked with 10% donkey serum for 30 minutes. After an overnight incubation with primary antibodies at 4°C, secondary antibodies conjugated with Alexa Fluor 488 or 594 (Invitrogen) were applied for 1 hour at room temperature. Cell nuclei were stained with DAPI (Sigma). The primary antibodies are as follows: anti-OCT4 (prepared by our lab), anti-SOX2 (prepared by our lab), anti-NANOG (R&D), anti-SSEA4 (Millipore), anti-SOX17 (R&D), anti-VIMENTIN (Abcam), anti-TUJ1 (Promega), anti-NESTIN (Millipore) and anti-PRRT2 (prepared by our lab).

RNA isolation, reverse transcription and quantitative PCR

Total cellular RNA was extracted by the Trizol Reagent (Invitrogen) and cDNA synthesis was performed using the SuperScript III First-Strand Synthesis System according to the manufacturer's guidelines. End-point RT-PCR was carried out using 0.2-1.0 μg of total RNA and oligo-d (T) primers in the PCR reaction systems (Life Technologies) reported previously [2]. Primer sequences for end-point RT-PCR are listed in Supplementary Table S1. Quantitative PCR was performed using the SYBR Premix Ex Taq (Takara Bio, Shiga, Japan) on Applied Biosystems 7900HT Fast Real-Time PCR System. Quantitative levels for all genes were normalized to the housekeeping gene *GAPDH* and assessed relative to the relevant control samples. Some sequences of the primers for PCR (pMXs-OCT4, pMXs-SOX2, pMXs-KLF4 and pMXs-C-MYC) have been reported previously [1, 3], other primer sequences for quantitative RT-PCR are listed in Supplementary Table S2.

Bisulfite sequencing PCR

Bisulfite genomic sequencing was carried out using the EZ DNA Methylation-Direct Kit (Zymo Research) as previously described [3]. Two μg of genomic DNA was treated according to the procedures of manufacture Active Motif. Primers specific to the human *OCT4* promoter were used to amplify genomic DNA sequences with Platinum Taq DNA Polymerase High Fidelity (Invitrogen) and PCR products were cloned into the pGEM-T easy vector (Promega). Individual clones were sequenced in the Invitrogen Company (Shanghai, China). Bisulfite primer information is presented in Supplementary Table S2.

Embryoid body (EB) formation

iPSCs were digested into small clumps and cultured on low-attachment dishes with the human EB medium for 9 days. Then formed EBs were collected and replated onto Matrigel-coated glass coverslips for additional 2 days, followed by immunofluorescence staining. The EB medium consists of DMEM (GIBCO) with 20% fetal bovine serum (FBS, Hyclone), 2 mM L-glutamine (GIBCO), 1% non-essential amino acid (GIBCO), 0.1 mM β-mercaptoethanol (Sigma), 100 U/ml penicillin and 100 μg/ml streptomycin (Sigma).

Teratoma formation

About 5x10^6 iPSCs were cultured in the presence of 10 μM Y27632 (Calbiochem) overnight. Then the cells were collected and injected intramuscularly into SCID mice anesthetized with isoflurane. At 8-10 weeks post-injection, teratomas were dissected, fixed overnight in 10% buffered formalin phosphate and embedded in paraffin. Sections were stained with hematoxylin and eosin (H&E) for further analysis. Experiments complied with all relevant institutional and national animal welfare laws, guidelines and policies.

Cell growth detection

The growth curve was detected using the Cell Counting Kit-8 (CCK-8, Dojindo) according to the manufacturer's recommended protocol. Cells were plated onto 96-well plate in a density of 1X10^5 cells per well, then cultured by treating with WST-8 [2-(2-methoxy-4-nitrophenyl)-3-(4-nitrophenyl)-5-(2,4-disulfophenyl)-2H- tetrazolium, monosodium salt], which could produce a water-soluble formazan dye upon reduction in the presence of an electron carrier, 10 μl of the CCK-8 solution was added to each well of the plate on day 1-10 and incubated for 4 hours in the incubator. The absorbance was measured at 450 nm and 600nm using the enzyme-labeled instrument (Promega).

Cell apoptosis detection by flow cytometry analysis

iPSCs cultured under the condition of step-wise neural induction were separately collected on days 0, 5 and 10. Assays were carried out using the Annexin V-PE/7-AAD Apoptosis Detection Kit (BD Biosciences) according to the manufacturer's manual. iPSC colonies were digested into single cells and stained with Annexin V-PE and 7-AAD for 15 minutes. Levels of cell apoptosis were analyzed using a BD Accuri C6 flow cytometer (BD Accuri Cytometers, Ann Arbor, MI, USA).

Immunofluorescence staining of the human fetal brain

A human fetal brain was obtained from an aborted fetal at autopsy within 3 hours of spontaneous abortion. The brain was examined with the informed consent of the donor and in accordance with the Tongji Hospital guidelines. Brain tissues were fixed in 4% PFA at 4 °C for 1 week and then cryoprotected in a sucrose gradient (15%-20%-30%) for 72 hours at 4 °C. Then samples were frozen in OCT on dry ice.

Immunofluorescence staining of the human fetal brain was performed on 10-20 μm serial coronal sections on glass slides. Some sections were subjected to an antigen retrieval protocol. Subsequently, cryosections were blocked for 1-2 hours in Tris-buffered saline with 2% Triton X-100 and 10% donkey serum. Cryosections were incubated with the primary antibodies (anti-PRRT2 and anti-NEUN) for 24 hours at 4 °C. Secondary antibodies against the appropriate species were incubated for 2-3 hours at room temperature, counterstained with DAPI for 15 minutes and coverslipped with the Organo/Limonene Mount (Sigma).

Western blot analysis

Human fetal tissues were isolated, then suspended in a lysis buffer supplemented with 1% protease inhibitor cocktail (Sigma), rotated and triturated at 4 °C. 10 to 20 μg of protein was separated on the 10% SDS-polyacrylamide gel, then transferred to a nitrocellulose membrane and probed with a primary antibody against PRRT2 overnight at 4 °C, followed by the incubation of horseradish-peroxidase-conjugated secondary antibody (Promega) at room temperature for 1-2 hours. Protein bands were visualized using ECL chemiluminescence analyzer (Thermo Scientific Pierce). GAPDH (Proteintech) were used as the loading control.

SUPPLEMENTARY REFERENCES

1. Li C, Zhou J, Shi G, Ma Y, Yang Y, Gu J, Yu H, Jin S, Wei Z, Chen F and Jin Y. Pluripotency can be rapidly and efficiently induced in human amniotic fluid-derived cells. Hum Mol Genet. 2009; 18:4340-4349.
2. Li L, Sun L, Gao F, Jiang J, Yang Y, Li C, Gu J, Wei Z, Yang A, Lu R, Ma Y, Tang F, Kwon SW, Zhao Y, Li J and Jin Y. Stk40 links the pluripotency factor Oct4 to the Erk/MAPK pathway and controls extraembryonic endoderm differentiation. Proc Natl Acad Sci U S A. 2010; 107:1402-1407.
3. Ma Y, Li CL, Gu JJ, Tang F, Li C, Li P, Ping P, Yang S, Li Z and Jin Y. Aberrant gene expression profiles in pluripotent stem cells induced from fibroblasts of a Klinefelter syndrome patient. J Biol Chem. 2012; 287:38970-38979.

SUPPLEMENTARY FIGURES AND TABLES

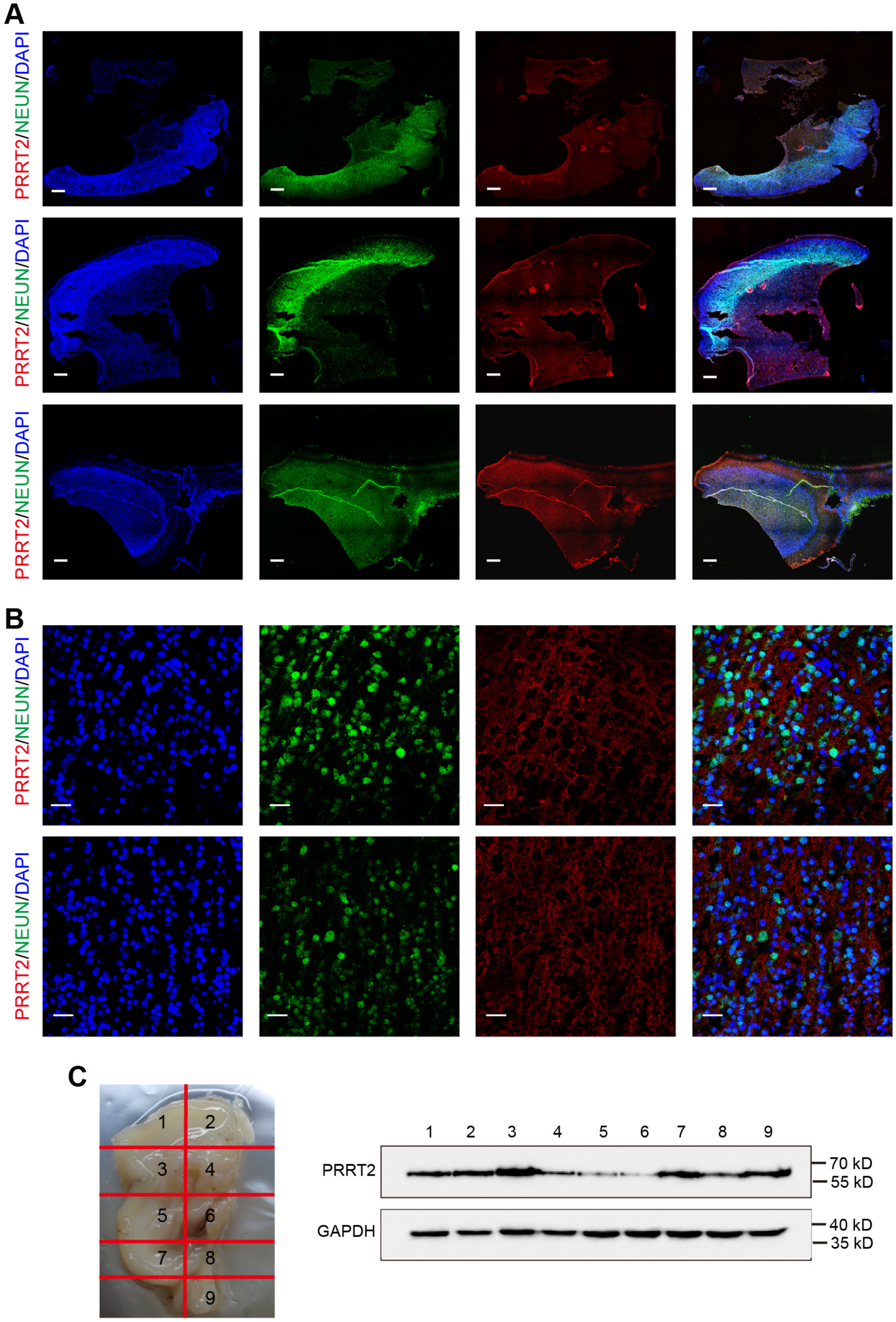

Supplementary Figure S1: The expression pattern of PRRT2 in human fetal brain. A. Immunofluorescence staining against PRRT2 in different sections of the human fetal brain. Scale bars, 500 μm. **B.** Partial enlarged images of the human brain sections. Scale bars, 25 μm. **C.** Western blotting analysis of PRRT2 in different sections of the human fetal brain. The corresponding anatomical regions separated from fetal brain are shown on the left.

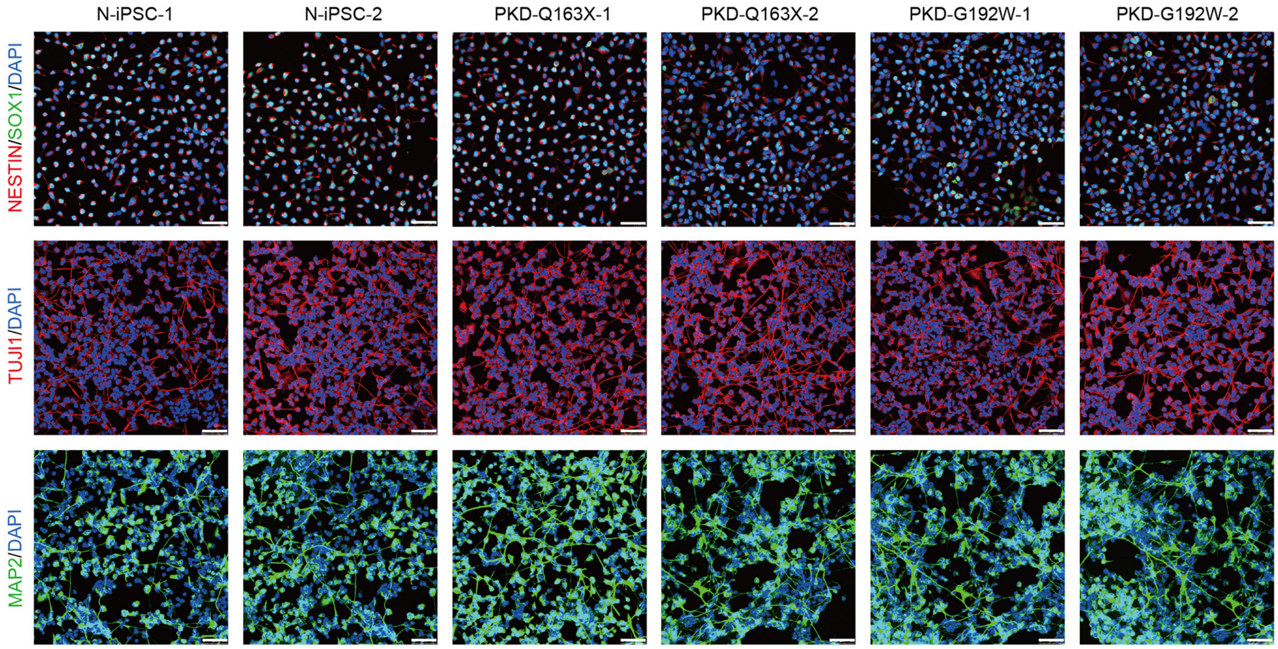

Supplementary Figure S2: Neural induction of PKD-iPSCs and normal iPSCs using a rapid single-step neural conversion approach. Immunofluorescence staining against neural lineage markers SOX1, NESTIN (NPC markers), TUJ1 and MAP2 (neuronal markers) in all PKD-iPSC lines and normal controls. Scale bars, 50 µm.

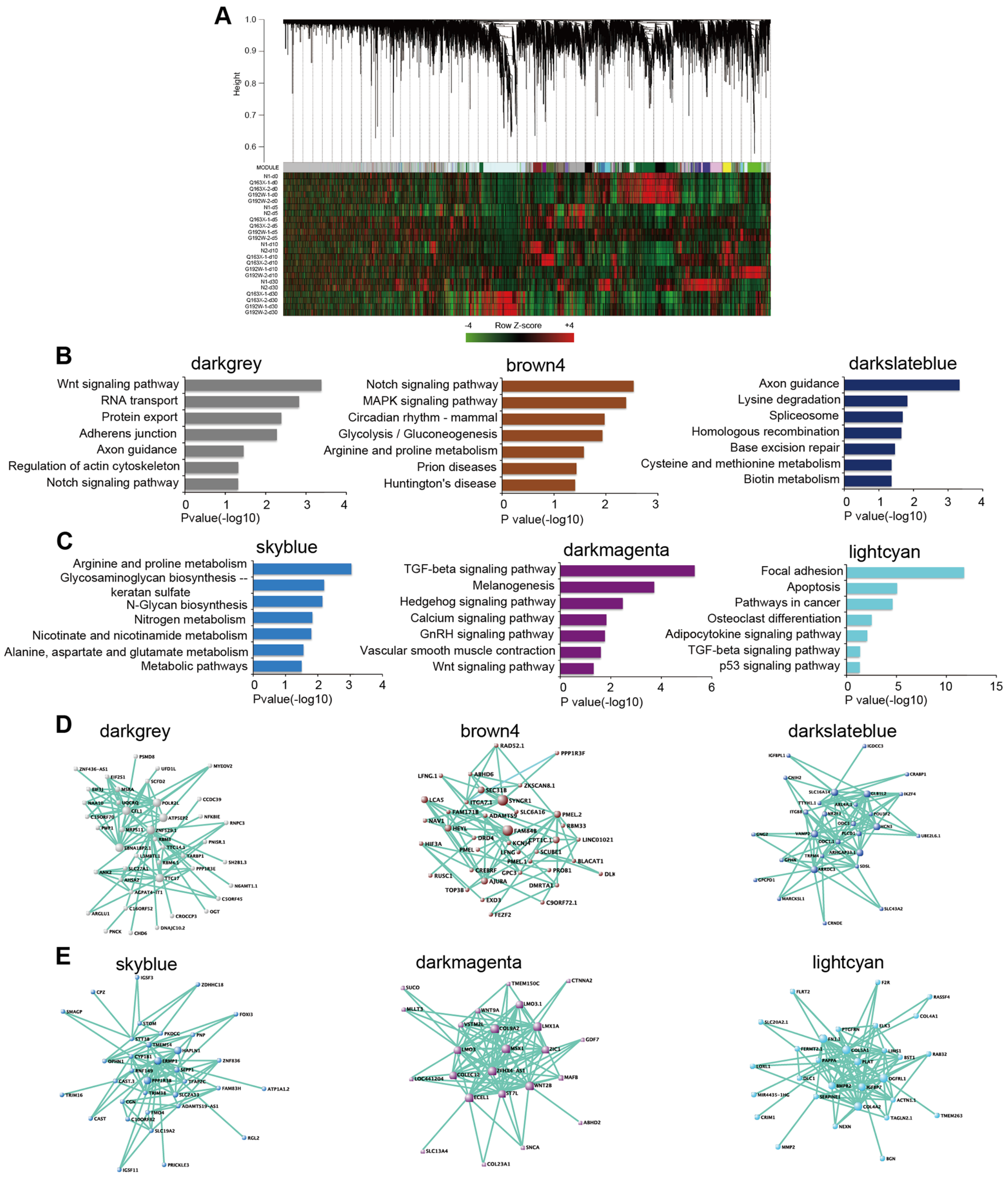

Supplementary Figure S3: WGCNA and functional analysis showing neural differentiation defects in PKD-iPSCs. A. WGCNA dendrogram indicates expression of different gene modules in all samples. Gene modules are labeled by different colors, which reveal highly positive correlated (red) or negative correlated (green) transcripts. **B-C.** KEGG pathways were identified in modules specific to different neural induction stages of PKD cells and normal cells, independently. The top seven significant KEGG pathways identified in each module are shown. Modules (darkgrey, brown4, darkslateblue) related with the normal group (N-d5, N-d10 and N-d30) are shown in (B), and modules (skyblue, darkmagenta and lightcyan) related with the PKD group (PKD-d5, PKD-d10 and PKD-d30) are shown in (C). The length of bars indicates $-\log_{10}$ P value of the Fisher's exact test. **D.** Hub-gene network of modules in (B) and (C) are visualized among the top 100 connections associated genes using VisANT. The size of the dots represents the hubness. The color of dots is consistent with its corresponding module.

Supplementary Table S1: Primers for end-point RT-PCR

Gene	Forward 5’ to 3’	Reverse 5’ to 3’
GAPDH	TCCACCCATGGCAAATTCC	TCGCCCCACTTGATTTTGG
Endo-OCT4	GACAGGGGGAGGGGAGGAGCTAGG	CTTCCCTCCAACCAGTTGCCCCAA
Endo-SOX2	GGGAAATGGGAGGGGTGCAAAAGA	TTGCGTGAGTGTGGATGGGATTGG
Endo-KLF4	ACGATCGTGGCCCCGGAAAAGGAC	TGATTGTAGTGCTTTCTGGCTGGG
Endo-C-MYC	CGAGAGGCAGAGGGAGCGAGCG	TTGAGGGGCATCGTCGCGGGAGG
NANOG	AGGCAAACAACCCACTTC	TCCCTGCGTCACACCATT
FGF4	GCGTGGTGAGCATCTTCG	GGTGACCTTCATGGTGGG
TDGF1	AGGAATTTGCTCGTCCATCTCGG	CGTGCTCACAGTTCCGTCCGTAG
LEFTY2	AGAGGGTGCTAAGAGGAAGGATGA	GAGTGGGCAGGGGTAGGTAGG
GDF3	ACCCCAGAAGTTCCAACCTG	AGAAAGCGAAGTACATTCCCG

Supplementary Table S2: Primers for quantitative RT-PCR and Bisulfite sequencing PCR (BSP)

Gene	Forward 5’ to 3’	Reverse 5’ to 3’
OCT4	ACATCAAAGCTCTGCAGAAAGAACT	CTGAATACCTTCCCAAATAGAACCC
NANOG	GAATGAAATCTAAGAGGTGGCA	CCTGGTGGTAGGAAGAGTAAAGG
SOX1	GGAATGGGAGGACAGGATTT	ACTTTTATTTCTCGGCCCGT
NESTIN	GAGAGGGAGGACAAAGTCCC	CCACTTCCTCAGACTGCTCC
TUJ1	AGTGTCCGCTCAGGGGCCTT	CCCTGCAGGCAGTCGCAGTTT
MAP2	AGGCGGAGCAGGGGAAGGAC	TCAGGACTGCTACAGCCTCAGC
PRRT2	GTCCAAACCAGAAGTGAGCA	AGGATGATGTAGTCCCGAGG
GAPDH	TCCACCCATGGCAAATTCC	TCGCCCCACTTGATTTTGG
OCT (BSP-O)	GTTAAGGTTAGTGGGTGGGATT	ATCACCTCCACCACCTAAAAA
OCT (BSP-I)	AGAGAGGGGTTGAGTAGTTTTTT	ACCTCCACCACCTAAAAAAAAC
